# Calcium Deregulation and Mitochondrial Bioenergetics in GDAP1-Related CMT Disease

**DOI:** 10.3390/ijms20020403

**Published:** 2019-01-18

**Authors:** Paloma González-Sánchez, Jorgina Satrústegui, Francesc Palau, Araceli del Arco

**Affiliations:** 1 Departamento de Biología Molecular, Centro de Biología Molecular Severo Ochoa, Consejo Superior de Investigaciones Científicas-Universidad Autónoma de Madrid (CSIC-UAM), 28049 Madrid, Spain; 2 Centro de Investigación Biomédica en Red de Enfermedades Raras (CIBERER), 28029 Madrid, Spain; fpalau@sjdhospitalbarcelona.org (F.P.); araceli.arco@uclm.es (A.d.A.); 3 Instituto de Investigación Sanitaria Fundación Jiménez Díaz (IIS-FJD), 28006 Madrid, Spain; 4 Instituto de Recerca Sant Joan de Déu and Hospital Sant Joan de Déu, 08950 Barcelona, Spain; 5 Division of Pediatrics, University of Barcelona School of Medicine, 08036 Barcelona, Spain; 6 Facultad de Ciencias Ambientales y Bioquímica, Centro Regional de Investigaciones Biomédicas, Universidad de Castilla la Mancha, 45600 Toledo, Spain

**Keywords:** GDAP1, recessive mutations, store operated calcium entry, mitochondrial location, calcium regulated cell respiration

## Abstract

The pathology of Charcot-Marie-Tooth (CMT), a disease arising from mutations in different genes, has been associated with an impairment of mitochondrial dynamics and axonal biology of mitochondria. Mutations in *ganglioside-induced differentiation-associated protein 1* (*GDAP1*) cause several forms of CMT neuropathy, but the pathogenic mechanisms involved remain unclear. GDAP1 is an outer mitochondrial membrane protein highly expressed in neurons. It has been proposed to play a role in different aspects of mitochondrial physiology, including mitochondrial dynamics, oxidative stress processes, and mitochondrial transport along the axons. Disruption of the mitochondrial network in a neuroblastoma model of *GDAP1*-related CMT has been shown to decrease Ca^2+^ entry through the store-operated calcium entry (SOCE), which caused a failure in stimulation of mitochondrial respiration. In this review, we summarize the different functions proposed for GDAP1 and focus on the consequences for Ca^2+^ homeostasis and mitochondrial energy production linked to CMT disease caused by different *GDAP1* mutations.

## 1. Introduction

Charcot-Marie-Tooth (CMT) disease is one of the most common inherited peripheral neuropathies, with an overall population prevalence estimated at 10-28/100,000 in Europe [1]. It is characterized by a degeneration of motor and sensory fibers that progresses slowly in a length-dependent manner. In general terms, CMT disease is classified into demyelinating (CMT1) or axonal (CMT2) forms, depending on the nerve conduction velocities in motor fibers of the median nerve [2]. More than 80 genes encoding proteins with very different functions have been linked to CMT disease and related neuropathies [3]. One of them is *ganglioside-induced differentiation-associated protein 1* (*GDAP1*), of which more than 80 mutations have been found in CMT patients [4,5,6].

Mutations in *GDAP1* show a wide range of severity and Mendelian heterogeneity [7], leading to several forms of CMT disease including: recessive axonal (AR-CMT2K) [6]; recessive demyelinating (CMT4A) [5]; recessive with intermediate clinical features (CMTRIA) [8]; and dominant with axonal features (CMT2K) [9,10].

GDAP1 is an outer mitochondrial membrane protein that is ubiquitously expressed with predominant expression in nervous tissues [6]. In the peripheral nervous system, GDAP1 has been found to be expressed mainly in axons rather than Schwann cells [11], but another study also reported the presence of GDAP1 in myelinating Schwann cells [12]. This protein contains two glutathione-S-transferase (GST) type domains [13] separated by an α-loop region, a C-proximal hydrophobic domain, and a C-terminal transmembrane domain which is critical for its targeting to the outer mitochondrial membrane [14]. GDAP1 has been proposed to play a role in a number of mitochondrial functions, including mitochondrial dynamics [12,15,16,17], redox processes [18,19] or mitochondrial transport, calcium homeostasis, and energy production [20,21].

Mitochondrial dysfunction has been shown to underlie numerous neurodegenerative diseases, including Alzheimer´s (AD), Parkinson´s (PD), Huntington´s disease (HD), and also other forms of CMT, such as CMT2A [22,23,24,25]. With this in mind, the implication of GDAP1 in CMT disease is not surprising, since GDAP1 is involved in many mitochondrial functions. At the present, the molecular pathogenesis of CMT disease caused by mutations in *GDAP1* remains unclear. One reason may be that the primary effect on mitochondria depends on the location of each mutation, and consequently on the specific protein domain and function affected. However, a cell-specific effect of each of these mutations cannot be excluded, given that GDAP1 is expressed in both peripheral neurons and in lower levels in Schwann cells [6,18].

## 2. Proposed Roles of Ganglioside-Induced Differentiation-Associated Protein 1 (GDAP1) in Mitochondrial Physiology

Different roles of GDAP1 have been proposed, most of which are related to mitochondrial functions. Several studies have suggested an involvement of GDAP1 in mitochondrial dynamics. Specifically, GDAP1 has been described as a fission factor whose activity depends on the fission factors Drp1 and Fis1 [15]. Expression of human GDAP1 in Fis1 mutants in *S. cerevisiae* recovers cell size and mitochondrial network morphology [26]. GDAP1 overexpression induced fragmentation of the mitochondrial network, however, *GDAP1* silencing enhanced the tubular aspect of mitochondria in some studies [12,27] but not in others [20]. In this context, the effect of missense mutations in *GDAP1* found in CMT patients on mitochondrial dynamics has been also addressed. Some studies have shown that overexpression of recessive *GDAP1* mutations was associated with a decrease in fission activity, but not all mutants reduced fission activity to the same degree [15]. However, other overexpression studies showed that these recessive mutations resulted in a fragmented mitochondrial network, with no differences compared to the wild type protein, whose overexpression also caused mitochondrial fragmentation [11,20]. Altogether, it is not clear how GDAP1 contributes to mitochondrial fission and this suggests that GDAP1 must have another function rather than mitochondrial fission.

When GDAP1 was identified, phylogenetic and structural analysis suggested that GDAP1 belongs to a GST family [6,13], but early studies did not show any evidence of glutathione-dependent activity [16,28]. However, a role of GDAP1 in regulation of cellular glutathione (GSH) content has been shown, in which it confers a protective response against oxidative stress conditions [18,19,29]. Thus, mouse hippocampal neural cells selected as resistant to oxidative stress showed increased GDAP1 levels and GDAP1 upregulation resulted in resistance against oxidative stress caused by GSH depletion. [18]. Loss of GDAP1 also caused mild oxidative stress in mouse hippocampal neural cells and in peripheral nerves in a mouse model of GDAP1-related CMT [18,19]. These studies suggest that GDAP1 acts as a sensor of the reduced to oxidized glutathione ratio (GSH/GSSG) participating in the release of an antioxidative response causing the recovery of GSH/GSSG. These results suggest a possible role of oxidative stress and chronic inflammation in the pathogenesis of GDAP1-related CMT, as shown to occur in other forms of CMT such as CMT1A [30] or CMT1C [31].

Another relevant finding to understand the cellular role of GDAP1 is the fact that it localizes to mitochondrial-associated membranes (MAMs) and interacts with trafficking-associated proteins [20]. MAMs are the place where physical communication between the endoplasmic reticulum (ER) and mitochondria takes place, with both organelles 10–30 nm apart [32,33]. This finding suggests participation of GDAP1 in the mitochondria-ER interface. Indeed, GDAP1 deficiency reduced contacts between mitochondria and ER in neuroblastoma cells, and overexpression of GDAP1 in HeLa cells increased co-localization between both organelles [20]. GDAP1 has also been involved in mitochondrial transport within the cell, based on the interaction found between GDAP1 and β-tubulin, and the trafficking-associated proteins, RAB6B and caytaxin [20]. The GDAP1 domain responsible for this interaction is the α-loop, which is located between the two GST domains. In CMT mutations of this domain, abnormal protein interactions between GDAP1 and the trafficking-associated proteins have been found [20]. Anomalous distribution of the mitochondrial network has also been found in mouse models lacking *Gdap1* [34]. Together, these results suggest that a failure in mitochondrial movement (toward the ER or other cell locations) that alters the distribution of the mitochondrial network may be relevant to the pathogenesis of GDAP1-related CMT.

In addition to its role in mitochondrial functions, GDAP1 is also expressed in peroxisomes, where it is involved in the regulation of peroxisomal morphology [17]. In N1E-115 cells, reduced levels of GDAP1 led to elongated peroxisomes, while overexpression triggered peroxisomal fragmentation [17]. Patients suffering from CMT disease caused by mutations in *GDAP1* show a wide range of clinical manifestations, with the recessive forms of the disease (CMT4A and AR-CMT2) much more severe than the dominant form (CMT2K) [7]. This fact points out that different molecular mechanisms may underlie the pathology of CMT due to different mutations. It is possible that the clinical phenotype of CMT disease caused by missense mutations depends on the specific protein domain affected and the function associated with it. The variability of clinical features in these patients can also be explained by the presence of modifier genes, whose influence on phenotype has been shown in several neurological disorders [35,36]. Recently, *juntophilin-1* has been described to act as a negative modifier of *GDAP1* [37], and the presence of the dominant *GDAP1* p.R120W mutation along with the *JPH1* p.R213P mutation in a CMT patient led to a more severe form of the disease [37].

## 3. Role of Mitochondrial Traffic and Location in Charcot-Marie-Tooth (CMT) Disease

Mitochondria carry out many essential functions in the cell; they are the major cellular source of ATP, obtained via oxidative phosphorylation, but also host several metabolic pathways, such as the citric acid cycle, β-oxidation of fatty acids, and pyrimidine metabolism, among others [38]. Mitochondria are the main site where reactive oxygen species (ROS) are generated [39], and they are also a key component of Ca^2+^ homeostasis in the cell, being able to modulate the dynamic of cytosolic calcium signals by buffering Ca^2+^ flux from the ER or the plasma membrane [40,41,42]. Ca^2+^ flux between the ER and mitochondria depends on the correct formation of MAMs [33]. Consequently, mitochondria are fundamental for several biological processes, such as cell proliferation and necrosis or apoptotic cell death [38].

Mitochondrial structure and localization are associated with their functionality. These two aspects become more important in cells with high energy requirements such as neurons, particularly those with long axons, in which mitochondrial positioning and turnover are more relevant due to the greater energy demand that occurs in axonal terminals. Specifically, synaptic transmission [43] and vesicle recycling [44] require larger amounts of energy. In sensory and motor neurons of the peripheral nervous system (which are the cells affected by CMT disease), the regions with the highest energy demands, and thus where mitochondria are concentrated, are: the distal region of the initial segment of the axon; the nodes of Ranvier; and the neuromuscular junctions (in motor neurons) or sensory end terminals (in sensory neurons) [45]. Interestingly, mitochondrial shape and localization are highly regulated and depend on mitochondrial dynamics, motility, and tethering, all processes that have been shown to be disrupted in *GDAP1*-related CMT models.

Defects in axonal transport or abnormal mitochondrial distribution in axons have been described in CMT arising from mutations in other genes, and other peripheral neuropathies involving mutations in mitochondrial proteins or in proteins with roles in mitochondrial transport [46,47]. For example, mutations in *Mitofusin 2* (*MFN2*), which encodes an outer mitochondrial membrane protein involved in mitochondrial fusion [48], are the primary cause of CMT2A, the most common form of the autosomal dominant axonal CMT disease [22]. Dorsal root ganglion (DRG) neurons expressing clinical *MFN2* mutations showed impairment in transport of mitochondria along the axons [49]. An abnormal mitochondrial distribution was observed in a different mouse model of CMT2A, which presented significant mitochondrial accumulation specifically in the distal axons [50]. Interestingly, the accumulation of axonal mitochondria in the distal part of the sural nerve has also been observed in CMT2A2 patients [51]. These results, together with a study that shows evidence for a role of MFN2 in mitochondrial transport through a direct interaction with the Miro/Milton complex [52], reinforce the hypothesis that a failure in mitochondrial transport along the axons is the main pathological mechanism involved in CMT2A2. Deficits in mitochondrial transport have been linked to other forms of CMT disease. Mutations in *HSPB1* and *HSPB8* cause CMT2F and CMT2L respectively, and in both situations a disruption of the cytoskeletal and axonal architecture, preventing mitochondrial movement along the axons, has been found [53,54,55,56]. Taking all this together, axonal transport as a therapeutic target in mitochondria-related CMT diseases has been proposed and it has been already addressed in different models of CMT2 associated with *HSPB1* mutations. Inhibition of histone deacetylase 6 (HDAC6), the major deacetylating enzyme of α-tubulin [57] has been tested in order to increase the acetylation of α-tubulin and so facilitate the binding of molecular motor proteins (dynein and kinesin family) to the microtubules [58]. Results obtained in several studies showed that the use of different selective inhibitors of HDAC6 reverted mitochondrial transport defects in both motoneurons obtained by induced pluripotent stem cells (iPSCs) differentiation from two patients with different *HPSB1* mutations [59] and in cultured DRG neurons expressing *HSPB1* mutations [60,61]. Remarkably, the restored motor and sensory problems have been observed in mutant *HSPB1* mice in vivo [61].

## 4. Ca^2+^ Deregulation in *GDAP1*-Related CMT Disease

**Figure 1:**
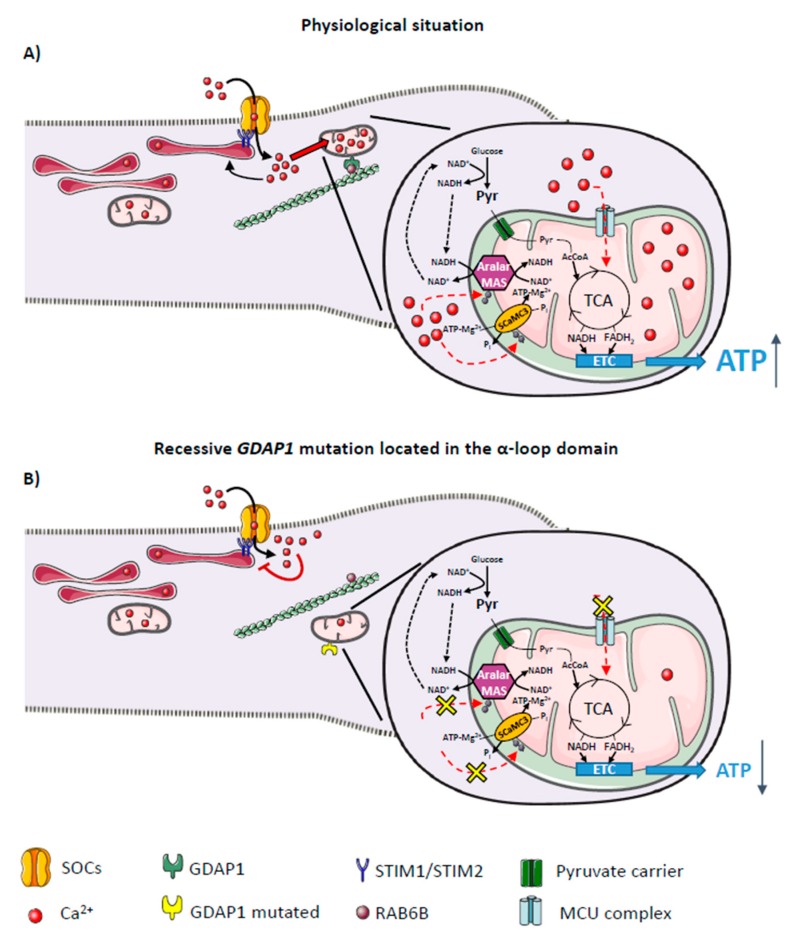
Effects of ganglioside-induced differentiation-associated protein 1 (GDAP1) on mitochondrial bioenergetic functions. (**A**) Interaction between GDAP1 and the trafficking proteins allows mitochondria to be located close to the plasma membrane after store-operated calcium entry (SOCE) activation, preventing its Ca^2+^-dependent inactivation. Ca^2+^ uptake by mitochondria facilitates SOCE but also regulates ATP production by oxidative phosphorylation. Ca^2+^-dependent regulation of OXPHOS involves two main mechanisms (dotted red arrows); (i) Ca^2+^ entry through the mitochondrial Ca^2+^ uniporter complex (MCUc) and the activation of dehydrogenases of the tricarboxylic acid cycle (TCA), and (ii) activation of the neuronal Ca^2+^-dependent mitochondrial transporters of aspartate/glutamate (Aralar) or ATP-Mg/Pi (SCaMC-3). Aralar activation increases Malate/Aspartate shuttle (MAS) activity, transferring reducing equivalents from NADH to mitochondria and thereby increasing pyruvate (Pyr) supply to mitochondria to enhance mitochondrial respiration. SCaMC-3 activation increases mitochondrial adenine nucleotide pool (solid and dotted black arrows); (**B**) Mitochondrial movement might be affected by recessive mutations located in the α-loop of GDAP1 causing the loss of interaction with trafficking proteins RAB6B and caytaxin which might affect the proper mitochondrial localization at the subplasmalemmal microdomains and disturb SOCE activity (red T bar). Subsequently, this will also impair mitochondrial bioenergetic functions by either decreasing Ca^2+^ uptake by MCUc and activation of matrix dehydrogenases, and/or by decreasing the activation of Ca^2+^-dependent mitochondrial transporters.

Impaired axonal mitochondrial movement and positioning found in different models of CMT disease [20,49,55,56] may affect multiple mitochondrial functions. *GDAP1* silencing in neuroblastoma cells or genetic disruption in mice caused a similar change in Ca^2+^ homeostasis: a failure to activate the store-operated calcium entry (SOCE) process [20,34], a cytosolic Ca^2+^ entry mechanism activated by a decrease in ER- Ca^2+^ levels [62]. Activation of SOCE is a dynamic process; store depletion is sensed by the ER stromal interaction molecule (STIM) proteins, STIM1 and STIM2, which oligomerize and migrate to the ER regions juxtaposed to the plasma membrane [63,64]. At the same time, proteins from the ORAI and transient receptor potential cation (TRPC) families which form the Ca^2+^ channel accumulate in the plasma membrane directly opposite of the STIM clusters [65,66]. Interaction between the channel proteins and the STIM clusters activates the store-operated calcium channels (SOCs), allowing Ca^2+^ influx into the cytosol [67]. This process is highly regulated by Ca^2+^, with two different mechanisms: a fast Ca^2+^-dependent inactivation mediated by Ca^2+^ near the mouth of the channel; and a slow Ca^2+^-dependent inactivation mainly performed by mitochondria [68]. The failure in SOCE found in GDAP1-deficient cells has been linked to a mislocalization of mitochondria at SOCE sites (Figure 1). These cells showed a smaller number of mitochondria close to the plasma membrane after SOCE activation, preventing proper mitochondrial Ca^2+^ uptake in the proximity of SOCs [20,21]. Mitochondrial Ca^2+^ uptake has been shown to facilitate SOCE by preventing its slow Ca^2+^-dependent inactivation in several cell types [68,69,70,71,72,73].

Interestingly, missense mutations in *GDAP1* seem to have different effects on Ca^2+^ homeostasis, depending on their pattern of inheritance [21]. Overexpression of dominant *GDAP1* mutations in *GDAP1*-silenced cells resulted in an increase in SOCE activity above control levels, while the effect of overexpression of recessive *GDAP1* mutations depended on the location of the mutation in the protein. Cells carrying recessive *GDAP1* mutations located in the GST or transmembrane domains showed no failure in SOCE, but those located inside the α-loop domain (the region involved in protein-protein interactions) mimicked the SOCE defect seen in GDAP1 deficiency [21]. Analysis of mitochondrial distribution in cells expressing these mutations may explain these differences in SOCE. Mitochondria carrying recessive *GDAP1* mutations located in the α-loop domain did not relocate to the plasma membrane under SOCE-activated conditions (Figure 1), a likely cause for SOCE impairment that is similar to previous descriptions in GDAP1-deficient cells [20]. However, in cells expressing dominant *GDAP1* mutations, mitochondria were already in close proximity to the plasma membrane in basal conditions [21]. This bias in basal mitochondrial distribution may be related to the increase in SOCE activity found in these mutants, whose Ca^2+^-inactivation may be prevented by resident mitochondria at the plasma membrane. 

The decrease in SOCE activity in recessive mutations in *GDAP1* α-loop domain has been linked to a decrease in ER- Ca^2+^ levels [21], consistent with the well-known function of SOCE in replenishing the intracellular Ca^2+^ stores [74]. Lower ER-Ca^2+^ levels have also been observed in GDAP1-deficient cells and in motor neurons from *Gdap1*-KO mice [34]. In neurons, ER- Ca^2+^ plays a relevant role in synaptic activity, regulating several processes including synaptic plasticity [75], spontaneous neurotransmitter release in synaptic boutons [76,77], and metabotropic glutamate receptor-dependent synaptic transmission [78]. Disruption of ER- Ca^2+^ levels also leads to activation of ER stress coping responses [79], such as the unfolded protein response (UPR), which activates apoptotic pathways if ER- Ca^2+^ homeostasis cannot be restored [80]. These processes may be triggered by disturbed Ca^2+^ homeostasis caused by the recessive mutations described above, but more studies are needed to elucidate the real consequences of the decreased ER- Ca^2+^ levels in neurons affected by CMT disease.

In conclusion, although alterations in SOCE have been described in dominant *GDAP1* mutations and recessive mutations located in the α-loop domain, the mechanism of CMT pathogenesis of each group of mutations seems to differ. These results suggest that decreased Ca^2+^ entry through SOCs and lower ER- Ca^2+^ levels, probably due to a failure of SOCE Ca^2+^ uptake caused by mislocalized mitochondria, may contribute to the pathogenesis of CMT patients carrying a recessive *GDAP1* mutation inside the α-loop domain (Figure 1). On the other hand, dominant mutations may act through a gain of function mechanism, which could be related to an increase in ROS production and apoptosis, as reported previously [15]. Interestingly, an increase in SOCE activity has been found to be detrimental in astrocytes in a mouse model of amyotrophic lateral sclerosis (ALS) [81], and in striatal neurons in a model of HD [82].

## 5. Neuronal Store-Operated Calcium Entry (SOCE) and Its Role in Neurodegenerative Diseases

Ca^2+^ is a major second messenger in cells [83,84] of critical importance in neurons since it is a key component of neurotransmission, synaptic plasticity, and gene transcription. Deregulation of Ca^2+^ homeostasis has been shown to be a common underlying mechanism of several major neurological disorders including PD [85], HD [86], AD [87,88], or ALS [89]. Therefore, control of Ca^2+^ homeostasis is essential for proper function of neurons. Neurons possess many different specialized Ca^2+^ channels and a collection of Ca^2+^ handling proteins, several of them specific to neurons [90,91]. SOCE is the main Ca^2+^ entry pathway in non-excitable cells [92], but its existence in neurons has been classically debated due to the presence of other major Ca^2+^ influx pathways [93,94]. However, SOCE activity has been found in different types of neurons both in the central [78,95,96,97,98,99] and peripheral nervous system [100]. Neuronal SOCE has been shown to perform different functions in resting neurons: refilling of Ca^2+^ stores [97]; control of spine morphology [101,102]; and regulation of neuronal gene expression [103]. Moreover, an increasing number of studies have also proposed a role of SOCE in neuronal synaptic activity by controlling Ca^2+^ dynamics [78,104,105,106] and different forms of synaptic plasticity [75,107,108,109], including long term depression [95].

Considering all these functions, it is not surprising that disruption of neuronal SOCE has been linked to major neurological disorders [110]. Different studies of familial AD caused by mutations in *presenilin* genes reported general alterations in Ca^2+^ homeostasis, and specifically an impairment in SOCE activity [101,111,112,113,114]. Additional evidence suggests that the molecular players of SOCE may also be involved in the pathogenesis of AD. Changes in STIM1 and STIM2, the sensors of intraluminal Ca^2+^ levels that transport information about Ca^2+^ load of the stores to channel proteins at the plasma membrane [67], have been found in cases of sporadic [101] and familial AD [115]. Decreased STIM2 levels caused an impaired neuronal SOCE in two different mouse models of familial AD, presenilin [101] and amyloid precursor protein knock-in (APPKI) mouse models [116], leading to a disruption of SOCE-Ca^2+^/calmodulin-dependent protein kinase II (CaMKII) pathway and finally, to mature spine loss. Importantly, overexpression of STIM2 in hippocampal neurons rescued the downstream signaling cascade and dendritic spine morphology in both presenilin [101] and APPKI mouse models [116]. The lack of STIM1 in differentiated neuroblastoma cells triggered a pathological increase in Ca^2+^ entry through L-type voltage-operated Ca^2+^ channels in response to depolarization [115], in agreement with the modulating role of STIM1 on these channels [117,118].

Similarly, a failure in SOCE activation may also play a role in the pathogenesis of PD. The loss of TRPC1 or STIM1, molecules involved in SOCE, has been linked to death of dopaminergic neurons due to an increase in L-type Ca^2+^ currents [119,120]. Moreover, skin fibroblasts from idiopathic and familial PD patients have been shown to have an impairment of SOCE activation not associated with reduction in Orai1, TRPC1, STIM1 or STIM2 expression, and depleted ER-Ca^2+^ stores [121].

On the contrary, hyperactive SOCE has been linked to HD. Studies using the transgenic YAC128 HD mouse model found an enhanced SOCE in striatal medium spiny neurons (MSNs) [122,123,124], which seems to compensate for an increase in Ca^2+^ leakage from the ER [122]. This abnormal activation of SOCE may underlie the striatal synaptic loss found in YAC128 mice [122]. In this HD mouse model, an increase in expression of the ER protein STIM2 has also recently been reported in cultured MSNs and the striatum. Accordingly, downregulation of STIM2 rescued the loss of dendritic spines [122]. Modulation of all molecular players of SOCE may have a neuroprotective role not only in HD [125], but also in other disorders where altered expression of these molecules has been found, such as epilepsy and traumatic brain injury (TBI). STIM1 and STIM2 are upregulated in the hippocampus of chronic epileptic mice and were found to be strongly expressed in hippocampal samples from a patient suffering from medial temporal lobe epilepsy [126]. This highlights a role for SOCE in somehow controlling neuronal network excitability, as proposed for dorsal horn neurons [127]. Expression of these two ER proteins was also found to be upregulated after induction of TBI in two different studies. Upregulation of STIM2, both in vitro and in vivo, triggered an increased SOCE, mitochondrial Ca^2+^ overload and deregulation of mitochondrial functions, such as ROS production. Interestingly, all these functions were recovered upon downregulation of STIM2 [128]. A different group showed STIM1 upregulation in response to neuronal injury, finding that *Stim1* knockdown improved neuronal survival and reduced neuronal apoptosis after TBI [105].

All these studies suggest that SOCE may be a common mechanism involved in Ca^2+^ deregulation in many neurological disorders, which may include CMT. However, the role and relevance of Ca^2+^ influx by SOCs in the neurons that are affected by CMT, i.e., the sensory and motor neurons of the peripheral nervous system, is yet to be determined.

## 6. Impact of Ca^2+^ Deregulation on Mitochondrial Bioenergetics in GDAP1-CMT Model

Mitochondria use Ca^2+^ uptake and release to modulate cytosolic Ca^2+^ signals, thereby regulating numerous processes in cells [42] including SOCE [129]. Mitochondrial Ca^2+^ handling is a highly regulated process [130]. Permeability of the outer mitochondrial membrane is attributed to the abundant expression of porins [131], whereas in the inner mitochondrial membrane, Ca^2+^ influx into the matrix is mediated by the mitochondrial calcium uniporter (MCU) [132,133]. The properties of the MCU are determined by several proteins that interact with it to form the MCU complex (MCUc) [42], including the Ca^2+^ sensitivity modulators MICU1, MICU2, and MICU3 [134,135,136], the essential MCU regulator (EMRE) [137], the dominant negative subunit MCUb [138] and the regulator (MCUR) [139]. Additionally, there are other routes of Ca^2+^ influx which are under debate [140]. The major Ca^2+^ efflux pathway is the Na^+^/Ca^2+^ exchanger (NCLX) [141]. Ca^2+^ uptake by mitochondria not only serves as a Ca^2+^ buffering system, but also as a pathway to regulate intrinsic functions, including the main mitochondrial task: ATP production by oxidative phosphorylation [142,143]. 

Ca^2+^ influx through SOCs has been shown to stimulate mitochondrial respiration coupled to ATP synthesis in human neuroblastoma cells [21]. Previous work in these cells found that SOCE stimulation by muscarinic receptor activation induced glucose uptake by activation of AMP kinase subsequent to CaMKKβ activation [144], which may provide respiratory substrates for SOCE-stimulated respiration. Importantly, SOCE-induced upregulation of mitochondrial respiration is impaired by GDAP1 deficiency or by the presence of recessive *GDAP1* mutations located inside the α-loop domain in these cells [21].

In excitable cells, the role of Ca^2+^ in tuning energy supply to demand has long been known [145,146,147]. Ca^2+^ regulates ATP homeostasis both by increasing ATP consumption via activation of several ATP-dependent processes, and by promoting ATP synthesis through activation of mitochondrial dehydrogenases and direct stimulation of oxidative phosphorylation (OXPHOS) [148]. Neuroblastoma cell experiments using BAPTA-AM, a calcium chelator that prevents Ca^2+^ signaling but not changes in workload [149], showed that Ca^2+^ signaling itself is needed to couple SOCE activity to mitochondrial respiration [21].

Ca^2+^-dependent regulation of OXPHOS involves two main mechanisms (Figure 1). On the one hand, Ca^2+^ entry into mitochondria through the MCUc [42] is known to activate different enzymes in the matrix, including the citric acid dehydrogenases: isocitrate dehydrogenase and α-ketoglutarate dehydrogenase, which directly bind Ca^2+^; and pyruvate dehydrogenase, which is activated by Ca^2+^-sensitive phosphatase activity [150,151]. Matrix Ca^2+^ has also been proposed to regulate both F_1_-F_0_ATPase activity, by reducing its resistance to ATP production, and some other respiratory complexes [148,151]. The second mechanism involved in Ca^2+^-dependent regulation of OXPHOS does not require Ca^2+^ entry into mitochondria, rather it depends on the activity of calcium binding mitochondrial carriers (CaMCs) (Figure 1). Two groups of carriers form this family: the aspartate-glutamate carriers (AGC); and the ATP-Mg/Pi carriers, also named SCaMC (for short CaMC) [152,153,154,155,156]. Both types of CaMCs are characterized by the presence of EF-hand domains that are oriented toward the intermembrane space and activated by cytosolic Ca^2+^ [157,158,159,160]; they both function along with the MCU to decode the Ca^2+^ signal into a mitochondrial response.

In neuroblastoma cells, a rise of mitochondrial Ca^2+^ levels has been shown upon SOCE activation, suggesting that both mechanisms, matrix and extramitochondrial Ca^2+^ signaling pathways, may be implied to boost respiration [21]. Establishing which of these mechanisms is involved will aid in identifying potential targets in GDAP1-related CMT diseases that may lead to a failure in mitochondrial energy metabolism. Disruption of any of these mechanisms has been found to impair mitochondrial bioenergetics in different cell types. For example, in cardiomyocytes, the MCU selectively mediates acute mitochondrial Ca^2+^ loading to augment ATP production [161,162], and a lack of MCU in skeletal muscle led to an impaired oxidative metabolism and a shift toward fatty acid metabolism [163,164]. On the other hand, in cortical neurons Aralar/AGC1 is required to adjust coupled respiration to ATP demand under different workloads by providing pyruvate to mitochondria [147,149,165]. These results indicate that each Ca^2+^-dependent regulation of OXPHOS may have different relevance depending on cell type.

In conclusion, in neuroblastoma cell lines, SOCE deregulation affects cellular respiration in cells carrying recessive *GDAP1* mutations inside the α-loop domain (Figure 1) [21]. This suggests that a failure in mitochondrial bioenergetics may contribute to the molecular pathogenesis of CMT disease caused by these mutations. It will be interesting to know whether a similar deregulation and failure in mitochondrial bioenergetics occurs in sensory and motor neurons in other types of GDAP1-related and other mouse models of CMT disease. 

## 7. Concluding Remarks

An impairment of axonal mitochondrial transport has been proposed to underlie the pathology of CMT due to mutations in different genes, as CMT2A2 [49], CMT2F [55,56], and CMT2L [53]. Studies of the effect of different CMT mutations in *GDAP1* showed changes in mitochondrial dynamics and distribution within the cell and may be across the axon, leading to a general disruption of the mitochondrial network [12,15,16,20,21]. As alteration of a mitochondrial process can also affect other cellular functions, it can be challenging to identify the true cause of the pathology. A failure in Ca^2+^ homeostasis as a consequence of mitochondrial mislocalization has been found in cells carrying dominant *GDAP1* mutations and recessive mutations located inside the α-loop domain (Figure 1), with an associated augmentation or decrease of SOCE activity, respectively [21]. Interestingly, no Ca^2+^ alterations have been found in recessive mutations located outside the α-loop domain of GDAP1, which may act by a completely different mechanism. Recessive *GDAP1* mutations inside the α-loop domain cause a failure in stimulation of mitochondrial respiration associated with an impairment in SOCE activity (Figure 1) [21], but this may also take place in other GDAP1-related CMT diseases. For example, the prolonged elevated ROS production described in dominant mutations [15] may trigger permanent damage to mitochondria, leading to an impairment in bioenergetics [166].

These results suggest that a failure in mitochondrial control of Ca^2+^ entry by SOCs could be a possible mechanism underlying the pathology of *GDAP1*-related CMT, although additional defects in other Ca^2+^ entry mechanisms, such as voltage gated Ca^2+^ channels, cannot be excluded, particularly those in neuromuscular junctions [167,168,169,170]. More studies are necessary to identify which Ca^2+^ mechanisms are altered in the affected neurons of *GDAP1*-related CMT patients and *Gdap1* mouse models in comparison with other mouse models of CMT.
